# Interactions between cancer cells and normal cells via miRNAs in extracellular vesicles

**DOI:** 10.1007/s00018-014-1811-0

**Published:** 2015-01-07

**Authors:** Nao Nishida-Aoki, Takahiro Ochiya

**Affiliations:** Division of Molecular and Cellular Medicine, National Cancer Center Research Institute, 5-1-1, Tsukiji, Chuo-ku, Tokyo, 104-0045 Japan

**Keywords:** Extracellular RNA, Extracellular vesicle (EV), Exosome, MicroRNA (miRNA), Cancer, Cancer therapy

## Abstract

MicroRNAs (miRNAs) exhibit many functions in biological activities. Recent studies have shown that miRNAs exist outside cells and are transferred between cells. Extracellular miRNAs are protected from ribonucleases found in body fluids through binding to specific proteins or by being encapsulated in lipid bilayer vesicles. Here, we review the mechanisms of the secretion and uptake as well as the functions of extracellular miRNAs, particularly those encapsulated in extracellular vesicles. Extracellular vesicles are related to cancer progression, and some miRNAs in extracellular vesicles are associated with cancer cells. We describe the transfer of cancer-related miRNAs between cancer cells and non-cancerous cells. Finally, we discuss the anticipated applications of miRNAs present in extracellular vesicles in diagnostics and therapeutics.

## Introduction

MicroRNAs (miRNAs) are small (18–25 nt) non-coding RNAs that repress gene expression through post-transcriptional degradation or translational repression [1]. The first identified miRNA, *lin*-*4*, was found in *Caenorhabditis elegans* in 1993 and was suggested to regulate transcription via an RNA–RNA interaction in the 3′ untranslated region (UTR) of *lin*-*14* mRNA [2]. miRNAs are conserved in a large number of species of living organisms [3, 4]. So far, 2,588 miRNAs have been identified in *Homo sapiens* [5].

The most fundamental function of miRNAs is RNA interference (RNAi), which is a mechanism of silencing gene expression by degrading mRNAs with a complementary sequence. The phenomenon of silencing by small RNA fragments was first observed in petunia plants in 1990 (referred to as ‘cosuppression’) [6, 7] and subsequently in *Neurospora crassa* in 1992 (referred to as ‘quelling’) [8], but the underlying molecular mechanism was unclear. In 1998, Mello and Fire first described the RNAi mechanism in studies on *C. elegans* using short interfering RNAs (siRNAs) [9, 10]. Since then, RNAi has been shown to be conserved in almost all species and to possess a common, fundamental mechanism of regulating gene expression. The discovery of RNAi has also accelerated molecular biology research by leading to the development of genetic technologies to knock down any desired gene.

The molecular mechanism of RNAi has been explained in detail previously (reviewed in [11]). In brief, a miRNA is processed from a stem-loop sequence of ~70 nt (pre-mir), which is excised from a longer primary transcript (pri-mir). The pre-mir is exported to the cytoplasm to be cleaved into double-stranded RNA to form the miRNA by Dicer, an RNase III enzyme. The double-stranded miRNA is incorporated into the miRISC complex, and the leading strand of the miRNA is retained in the complex. miRNAs bind to a perfect complementary or a nearly perfect complementary sequence within a mRNA, and the sequence is cleaved by the miRISC complex.

Our knowledge of the importance of miRNAs in various bioactivities has been increasing. In addition to RNAi, miRNA regulates transcriptional levels in another manner: through inhibition of translation by binding to the 3′-UTR [12] and 5′-UTR of mRNAs [13]. Recent studies have also shown that miRNAs recognize coding regions [14]. Because miRNAs recognize sequences with imperfect pairings, a single miRNA can target a broad range of mRNAs with nearly complementary sequences, thereby increasing its impact on transcriptional regulation. Furthermore, miRNAs are involved in gene silencing through chromatin remodeling [15], which implies that miRNAs also play a role in epigenetic regulation. miRNAs have also been suggested to regulate DNA methylation and telomere recombination [16]. Through these mechanisms, miRNAs are involved in many physiological roles and developmental processes as well as in the progression of some diseases, such as cancer [17]. A reduction of tumor-suppressive miRNAs or overexpression of tumor-promoting miRNAs has been observed in cancer progression [18].

These post-transcriptional regulation and the other functions of miRNAs are all carried out in the cytoplasm or nuclear envelope; that is, inside the cell. However, miRNAs have also been found outside cells in various body fluids, such as serum, saliva, urine, breast milk, and tears [19–22]. These miRNAs are spontaneously secreted from cells. Moreover, these extracellular miRNAs function in recipient cells, indicating roles in cell–cell communication. Extracellular miRNAs also have been suggested to be related to several diseases, including cancer. In this review, we describe cell–cell communication between cancer cells and non-cancerous cells via miRNA packaged in extracellular vesicles and potential applications for therapeutic strategies.

## Extracellular miRNAs

RNAs have been discovered outside cells, even though RNAs have generally been thought to function inside cells. The following types of RNA have been found in extracellular environments: mRNA, tRNA, miRNA, small interfering RNA (siRNA), and lncRNA; however, almost no rRNA was detected in studies on a mast cell line and glioblastoma [23, 24]. In humans, miRNAs have been found in several body fluids, such as blood plasma [22], urine [25], saliva [26], semen [27] breast milk [28], and cerebrospinal fluid [29]. The detection of miRNAs outside cells drew interest because some body fluids, such as saliva, milk, and urine, contain a high concentration of nucleases [30]. Indeed, miRNAs from serum were shown to remain stable after several treatments that degraded most RNAs [31]. These reports suggested that extracellular miRNAs are protected from ribonucleases in some manner. Several mechanisms for preventing the degradation of miRNAs have been demonstrated. One possible mechanism used to protect miRNAs from degradation is RNA-binding proteins, such as Argonaute 2 (Ago2) [32], other Ago proteins (Ago1 [33], Ago3, Ago4 [34]), and nucleophosmin 1 [35]. High-density lipoproteins (HDLs) and low-density lipoproteins (LDLs) also interact with and transfer miRNAs to cells [36]. An alternative mechanism used to protect miRNAs from degradation involves the packaging of miRNAs in small, lipid bilayer vesicles, referred to as extracellular vesicles (EVs) [23]. Previously, we have found that approximately 30 % of extracellular miRNAs are packaged into EVs in both cell culture and sera (unpublished). These two mechanisms are independent according to a study showing that Ago2 transfers miRNAs independent of EVs based on differential centrifugation and size-exclusion chromatography analyses [32]. However, some reports discuss the possibility that extracellular miRNAs associated with proteins are not spontaneously secreted but represent cellular residue released from dead cells [34]. In addition, evidence of the uptake of protein-associated miRNAs and their function in the recipient cells remains scarce. The mechanisms and functions of protein-bound miRNAs should be elucidated to demonstrate the biological role of miRNAs associated with protein and to determine whether extracellular miRNAs protected by proteins and miRNAs encapsulated in EVs exhibit different secretion pathways and functions. This review primarily addresses miRNAs secreted in vesicular form, for which the mechanism has been more clearly elucidated.

## Extracellular vesicles (EVs)

Extracellular vesicle is a comprehensive term for lipid bilayered vesicles that exist outside cells [37]. EVs include apoptotic bodies (800–5,000 nm diameter), microvesicles (50–1,000 nm diameter), and exosomes (40–100 nm diameter) [38, 39]. These EVs have different origins and properties. Apoptotic bodies are released from the cell surface during the process of apoptosis [40]. Microvesicles are also formed by budding directly from the cellular membrane [41], whereas exosomes first bud into a multivesicular body (MVB), which is an intracellular compartment, and are then secreted to the extracellular milieu [39]. Different RNA profiles have been demonstrated for each type of vesicle [42]. Apoptotic bodies primarily contain rRNA, and microvesicles carry little RNA, except for microvesicles from TF-1 cell culture. Exosomes contain mRNA and miRNA, but little rRNA [42]. Proteins on the surface are also different among the three vesicles. CD9 and CD63 are recognized as potential markers of exosomes, ARF6 and VCAMP2 are proposed as microvesicle markers, and TSP and C3b are accepted as markers for apoptotic bodies (reviewed in [43]). With respect to lipid compositions, exosomes have low phosphatidylserine (PS) at outer leaflet, while microvesicles and apoptotic bodies contain high amount of PS [44]. Enrichment of ceramide is one of the characteristics of exosomes [45].

However, we must consider that the currently used techniques are inadequate for collecting each type of EV separately [46]. In addition, the nomenclature of exosomes is confusing [37]. In 1983, two groups reported that reticulocytes release transferrin receptors associated with small vesicles [47, 48]. These authors showed that the vesicles were formed inside multivesicular endosomes. The term ‘exosome’ was subsequently applied to the vesicles in 1987 [49]. However, in 1981, microvesicles released to cell cultures from the cell membrane of normal and neoplastic cell lines with 5′-nucleotidase activity were referred to as ‘exosomes’ [50]. ‘Exosome’ is also used to refer to the exoribonucleases identified in *Saccharomyces cerevisiae* [51]. The International Society of Extracellular Vesicles recommended the nomenclature ‘EVs’ as a generic term for all secreted vesicles, and the terms should be defined clearly before use [37]. In this review, we use the term ‘EVs’ for collective extracellular vesicles, and ‘exosomes’ for extracellular vesicles which are formed by budding into MVBs.

EVs have long been thought of as no more than a garbage can for cells to discard unwanted components [52]. mRNAs, DNAs, and proteins have been detected within these vesicles [53], and miRNAs were discovered inside EVs decades later, in 2007 [23]. Valadi et al. identified miRNAs as well as mRNAs inside EVs from mouse and human mast cell lines and proved that these nucleic acids are functional in the recipient cells. These findings drew much attention to EVs, and miRNAs were recognized as a new regulatory component in gene expression. Based on the reports accumulated to date, almost all cells are now considered to release EVs [23].

## Secretion of miRNAs packed in exosomes

**Fig. 1:**
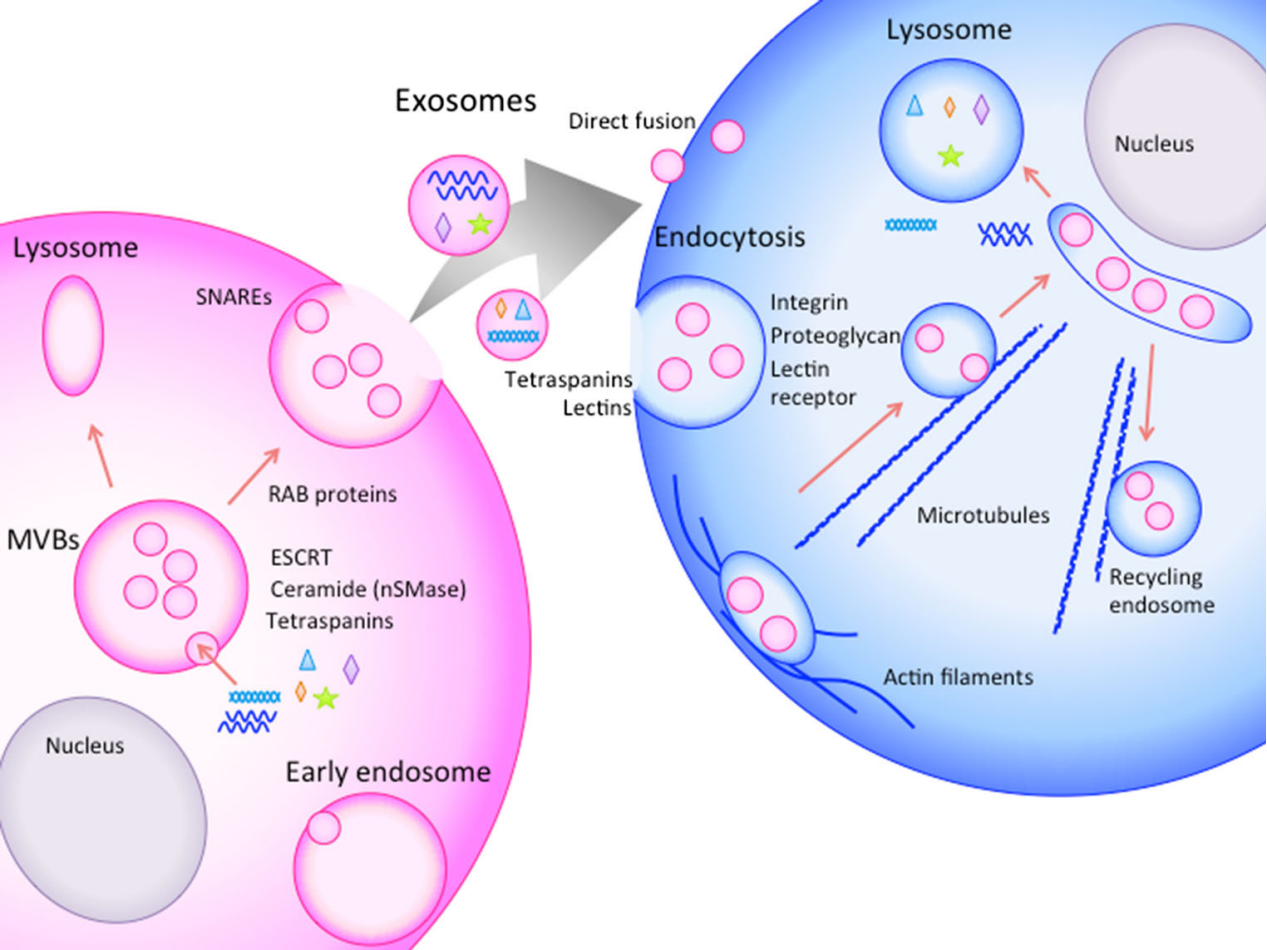
Schematic representation of the secretion, transfer, and uptake of exosomes. Exosomes are formed by budding into early endosomes to form MVBs. The internal vesicles are secreted outside the cells via MVB fusion with the cellular membrane. The released exosomes are taken up by neighboring cells or distant cells. Exosome internalization occurs through either direct fusion or endocytic pathways. The exosomes are carried outside the cells for lipid recycling

Studies examining the molecular mechanisms of exosomes release from one cell and their uptake by other cells are ongoing (described in Fig. 1). Exosomes are formed by budding into early endosomes to form MVBs. MVBs fuse with the cellular membrane or lysosomes. When fused with the plasma membrane, internal exosomes are released outside the cell [54].

The mechanism underlying the formation of exosomes into MVBs is controversial. The biogenesis of exosomes was first suggested to be associated with the endosomal sorting complex required for transport (ESCRT) [55]. ESCRT-related proteins, such as HRS (ESCRT-0), TSG101 (ESCRT-I), CHMP4 (ESCRT-II), ALIX and VPS4 (ESCRT-III), have been shown to be recruited to the cytosolic sides of MVBs to form exosomes (summarized in [56]). However, a recent report demonstrated that mammalian cells without key ESCRT components could still form MVBs [57]. Some studies have revealed some exosome formation that is independent of ESCRT, and this process depends on several molecules, such as ceramide [45] or tetraspanins [58]. Neutral sphingomyelinase 2 (nSMase2), a rate-limiting enzyme in ceramide biosynthesis [59], affects the secretion of miRNAs [60]. The nSMase2-dependent transfer of miRNAs via exosomes contributes to cancer metastasis in mouse breast cancer [59]. Tetraspanins are enriched on the surface of exosomes, and some of these proteins, such as CD9, CD63, and CD81, are also used as marker proteins of the exosome. Bone marrow dendritic cells from CD9 knockout mice were incapable to secrete exosome [61], while CD81 seemed not effect exosome secretion in lymphocytes [62] (reviewed in [58]). CD63 has been suggested to be instrumental in the formation of internal vesicles in melanocytes [63].

MVBs are sorted in two ways: through fusion with lysosomes or through fusion with the cellular membrane to secrete exosomes [56]. During the secretion steps, RAB proteins (RAB11, RAB27, and RAB35) have been shown to be involved in the transport of MVBs to the plasma membrane and in exosome secretion [56]. In addition, SNAREs have been suggested to be involved in MVB fusion with the plasma membrane [46].

Within exosomes from human, more than 700 miRNAs have been identified to date, together with thousands of proteins, DNAs, and mRNAs (Vesiclepedia and EVpedia) [64–67]. Several studies using microarrays or next-generation sequencing have identified a dozens of non-coding RNAs in exosomes froma single cell line [68, 69]. However, based on the size of exosomes (~100 nm), not all miRNAs are packaged into a single exosome. Exosomes from the same cell line are likely to exhibit different contents [70, 71].

The miRNA concentration within exosomes appears to partially depend on the concentration inside the cell. For example, exosomes secreted from cells overexpressing a certain miRNAs contain more of the overexpressed miRNAs [72]. However, many reports have shown that the profiles of miRNAs in exosomes are different from those of exosome-producing cells, suggesting that miRNAs are selectively packaged into exosomes. For example, although rRNA accounts for the majority of the RNA in a cell, almost no rRNA was detected in studies examining exosomes from a mast cell line and glioblastoma [23, 24]. The first paper detecting miRNAs in exosomes showed that only a fraction of miRNAs from all miRNAs expressed in the cells were detected in exosomes [23]. Moreover, some miRNAs were found at higher levels in exosomes than in the cells [23]. In MCF7 cells (breast cancer cells), only 2 % of many miRNAs including miR-720, the most abundant miRNA in the cell, were released into the environment, whereas miR-451 and miR-107 were secreted at higher levels [73]. However, the molecules responsible for selectively packaging miRNAs into exosomes remain to be identified. Villarroya-Beltri et al. [74] discussed that sumoylated form of the heterogenous ribonucleoprotein A2B1 (hnRNPA2B1) would load miRNAs into exosomes through EXOmotif (GGAG) in the miRNAs. The mechanisms of miRNA secretion can be applied to design exosomes that contain specific miRNAs. This technique would enable us to produce exosomes carrying tumor-suppressive miRNAs for cancer therapy.

## Uptake of exosomes into target cells

Released exosomes may be taken up by nearby cells, or they may reach cells at a distance through the blood or lymph system. However, the mechanism of uptake is more obscure than the secretion mechanism. The mechanisms of uptake suggested to date include endocytic pathways, such as endocytosis, phagocytosis and macropinocytosis, and direct fusion with the cellular membrane [54, 75–78] (Fig. 1). Escrevente et al. [79] showed that proteinase K treatment of exosomes significantly reduced uptake by ovarian cancer cells. The results indicate that surface proteins on exosomes take part in the uptake of exosomes. Mulcahy et al. suggested in a review that proteins on the surface of exosomes such as tetraspanins and lectins are involved in exosome uptake. They also suggested that integrins, proteoglycans, and lectin receptor proteins on the cell surface may serve as receptor of exosome surface protein for uptake (reviewed in [80]).

Concerning the direct fusion mechanism, Parolini et al. showed, using fluorescently labeled exosomes from melanoma cells, that lower pH, which is linked to tumor malignancy, increases exosome release and uptake. The authors indicated that a high rigidity and a high content of sphingomyelin/ganglioside GM3 and caveolin-1 in exosomes are responsible for accelerated fusion under a low pH [78].

Endocytic uptake was first suggested in dendritic cells [81]. Some reports have identified the molecules involved in exosome uptake through endocytosis. Dynamin 2 (Dyn2), which is necessary for clathrin- and caveolin-dependent endocytosis, was shown to be essential for the internalization of exosomes, suggesting that exosomes are taken up via clathrin- or caveolin-dependent endocytosis [75]. Heparan sulfate proteoglycans (HSPGs) have been suggested to serve as the receptors of cancer cell-derived exosomes in glioblastoma patients [82]. This finding suggests a mechanism similar to a virus entering a cell, as HSPGs are also known to be involved in viral entry [82]. In addition, exosomes from glioblastoma cells are internalized into HUVECs through lipid raft-mediated endocytosis and are negatively regulated by caveolin-1 (CAV1), although CAV1 was not found to co-localize with exosomes. CAV1 was suggested to have negative effect on exosome internalization through extracellular signal-regulated kinase-1/2 (ERK1/2)- and heat shock protein 27 (HSP27) signaling [83].

Tian et al. [84] visualized the cellular uptake and intracellular trafficking of exosomes in vitro at a single-particle level and indicated that exosomes may enter cells through endocytic pathways. First, exosomes are transported along the cell periphery, potentially by actin filaments. Exosomes are then carried to perinuclear regions, possibly on microtubules via dynein-dependent transport, to be trapped in large vesicles. At the same time, exosomes are transferred from the perinuclear space to the cell surface, which may occur along microtubules via kinesins for lipid recycling. The proteins in exosomes were observed to be released from exosomes within 3 h after endocytosis and through the fusion of endosomes or late endosomes in an acidified environment. Then, the proteins are sorted into lysosomes. When and how miRNAs are released to functionally regulate transcription in the cytosol has not been demonstrated.

Whether exosomes are selectively taken up by certain cells has been investigated. Many reports have suggested that exosomes are delivered specifically to cells [85]. Unidirectional transfer of miRNAs from T cells to antigen-presenting cells via exosomes has been reported [72]. Proteins at the surface of exosomes seemed to be involved in target-specific internalization into cells. However, the primary mechanism by which exosomes selectively interact with target cells has not been elucidated.

After exosomes are uptaken by recipient cells, exosome-transferred miRNAs function in transcriptional regulation in recipient cells [53]. In 2010, four independent groups showed that miRNAs transferred to recipient cells function to repress the expression of target genes [60, 86–88]. As exosomes are thought to be secreted from all types of cells, these vesicles are likely to be responsible for important physiological functions through cell–cell communication. Indeed, reports have shown that exosomes are involved in physiological functions, for example, in immune response (T cells, B cell, and dendritic immune cells) [28, 72] and neuron-glia communication [89]. However, the molecular mechanisms of how exosomes act in physiological functions have remained elusive because of the limits of the experimental methods for detection and for tracking exosomes from specific cells in vivo.

## Cancer-promoting EVs and their miRNAs

The expression levels of miRNAs are dynamically altered in cancer cells compared to normal cells [86]. Many miRNAs have been reported to play roles in both accelerating and inhibiting of cancer progression [20, 87]. These cancer-related miRNAs have been detected not only in cancer cells but also in cancer-derived EVs (reviewed in [52]). Indeed, increasing evidences suggested that EVs contribute to malignancy in cancer [90]. Cancer cells release more EVs than normal cells [91, 92]. Tumor-derived EVs are recognized as antigens by dendritic cells, inducing antitumor immune responses [70]; at the same time, cancer-derived EVs are associated with tumor malignancy and progression. Inhibition of EV secretion using a short hairpin RNA targeting RAB27A, a protein involved in intracellular trafficking of EVs, led to poor development of subcutaneously injected 4T1 mouse mammary tumor cells [93]. Furthermore, inhibition of EV secretion through nSMase2 knockdown inhibited vascular formation in primary tumors, and the opposite response was observed after nSMase2 overexpression [59]. Here, we describe some of the cancer progression-related miRNAs detected in EVs thus far (Table 1).

**Table 1:** Cancer-related miRNAs in EVs

Function	Secretion cells	Target cells	miRNA	miRNA targets	References
Angiogenesis	Mouse mammary breast cancer (4T1)	Endothelial cells (in vivo, in vitro)	miR-210		[52]
	Melanoma	Endothelial cells	miR-9	SOCS5	[96]
	Breast cancer cells (MDA-MB-231)	Endothelial cells	miR-105	ZO-1	[97]
Suppression of anti-tumor genes	Breast cancer (MCF-7) Glioblastoma malignant ascite		miR-21	PTEN PDCD4	[73] [98] [101]
Release of tumor-suppressive miRNAs	Metastatic gastric cancer (AX-P7a)		Let-7	RAS, HMGA2	[103]
Dormancy	Bone mallow mesenchymal stem cells	Breast cancer cell (MDA-MB-231)	miR-23b	MARCS	[105]
Transfer of drug resistance	Drug-resistant breast cancer (MCF-7)	Drug sensitive breast cancer (MCF-7)	miR-100, miR-222, miR-30a	miR-222, 30a: pathways for cancer miR-222: cell cycle, PTEN	[106]
Immune suppression	HEK-293	Murine macrophage	miR-21, miR-29a	Directly bind Toll-like receptor	[109]
Promote invasiveness	IL-4-activated M2 macrophages	Breast cancer cells (SKBR3 and MDA-MB-231)	miR-223	MEF2C	[110]
Metastasis	Colorectal cancer		miR-21	PDCD4	[100]
Premetastatic niche formation	Renal cancer stem cells	Lung cells (in vivo)	miR-200c, miR-92, miR-141, miR-29a, miR-650, miR-151		[112]
	Metastatic rat adenocarcinoma	Lymph node stroma cells, lung fibroblasts	miR-494, miR-542-3p	Cadherin-17	[113]
Tumor suppressive	Prostate epithelial cells (PNT-2)	Prostate cancer cells (PC-3M)	miR-143	Suppression of KRAS and ERK5	[114]

### Tumor proliferation and angiogenesis

Communication between tumor cells and their surrounding microenvironment has a large impact on tumor proliferation [94]. Cancer cells modify their microenvironment by transferring miRNAs that are suitable for their growth via EVs. miR-210 is a major cancer-related miRNA that is induced in hypoxia [95]. In EVs from highly metastatic breast cancer, miR-210 was found to be enriched and to be responsible for the induction of angiogenesis in the tumor microenvironment [59]. Endothelial cells that received tumor-derived EVs underwent vascular formation [59]. miR-9 is delivered by melanoma-derived EVs into endothelial cells and promotes endothelial cell migration and angiogenesis by reducing SOCS5 and activating the JAK-STAT pathway [96]. miR-105 in EVs from breast cancer cells targets the tight junction protein ZO-1, leading to destruction of vascular endothelial barriers and promotes metastasis [97].

Cancer cells release miRNAs to decrease the expression of tumor suppressor genes. miR-21, which contributes to the development of cancer, has also been identified in cancer-derived EVs [98]. miR-21 represses *PTEN* [99] and *PDCD4* [100], which are known tumor suppressor genes. miR-21 has been detected in EVs from breast cancer cells [73], glioblastoma [98], and malignant ascites [101]. Let-7 miRNA, which was found to play tumor-suppressive roles in early studies [102], accumulates in secreted EVs from a metastatic gastric cancer cell line, AZ-P7a, compared with low-metastatic cells and other types of cancer cell lines [103]. Let-7 miRNA downregulates oncogenes such as *RAS* and *HMGA2*, suggesting that AZ-P7a cells release let-7 miRNA via EVs to maintain their oncogenesis [103].

### Tumor dormancy

Breast cancer is known to present especially late recurrence after 10–20 years [104]. EVs from bone marrow mesenchymal stem cells (BM-MSCs) are thought to enable breast cancer cells in the bone marrow to be dormant for 10 years, which is considered the main cause of breast cancer reoccurrence after a long period [105]. miR-23b in EVs from BM-MSCs were suggested to suppress *MARCS*, which encodes myristoylated alanine-rich C kinase substrate and promotes cell cycling and motility [105].

### Transfer of drug resistance

In cancer, EVs also contribute to malignancy by enhancing the drug resistance of the recipient cells. Using the MCF-7 cell line, Chen et al. showed that EVs from drug-resistant breast cancer cells enhance drug resistance in proximate cells by modulating the cell cycle and drug-induced apoptosis. Through microarray analyses and confirmation via quantitative real-time PCR, miR-100, miR-222, and miR-30a were shown to be increased in EVs from drug-resistant breast cancer cells, suggesting that these miRNAs contribute to the transfer of drug resistance [106].

### Suppression of the immune system

During cancer progression, the immune system attempts to remove cancer cells. However, many reports have shown that EVs released from cancer cells can repress the immune system through multiple pathways [107, 108]. In most cases, proteins within and on the surface of EVs play roles in suppression; however, miR-21 and miR-29a in EVs derived from lung cancer cells were observed to be able to bind to Toll-like receptors on surrounding immune cells to trigger an inflammatory response, leading to the secretion of pro-metastatic inflammatory cytokines [109]. Cancer cells also take advantage of immune cell-derived EVs. Tumor-associated macrophages were shown to release EVs containing miR-223, which is specific for IL-4-activated M2 macrophages. miR-223 transferred to breast cancer cells targets the 3′-UTR of *MEF2C*, a myocyte enhancer factor. The reduction of *MEF2C* is linked to nuclear accumulation of the function of β-catenin to promote the invasiveness of the SKBR3 and MDA-MB-231 breast cancer cell lines [110].

### Tumor metastasis

Extracellular vesicles derived from cancer cells have also been suggested to contribute to metastasis by modifying the environment of distant organs. To form colonies in metastatic organs, cancer cells must overcome several barriers, one of which is surviving and proliferating in metastatic organs. Tumor cells have been suggested to condition the metastatic area beforehand using EVs to form an environment that is suitable for growing cancer cells. The microarea conditioned by EVs is referred to as the premetastatic niche [111]. Some miRNAs involved in the formation of the premetastatic niche have been identified. In renal cancer stem cells, several miRNAs (miR-200c, miR-92, miR-141, miR-29a, miR-650, and miR-151) found in EVs are related to the formation of the lung premetastatic niche and lead to tumor invasion and metastasis, in addition to inducing angiogenesis [112]. In EVs from metastatic rat adenocarcinoma, miR-494 and miR-542-3p were shown to be increased and to prepare premetastatic organ stroma cells by affecting proteases, adhesion molecules, chemokine ligands, cell cycle- and angiogenesis-promoting genes, and the oxidative stress response [113].

## EVs from non-cancerous cells with tumor-suppressive effects

Many reports have shown that EVs from cancer cells contribute to cancer progression. However, normal cells also release EVs that play inhibitory roles in cancer progression, although few transferring tumor-suppressive miRNAs have been reported to inhibit the proliferation of cancer cells. Epithelial prostate cells, PNT-2, secrete EVs containing miR-143, which repressed proliferation of prostate cancer cells, PC-3M [114]. Other tumor-suppressive miRNAs, including miR-15a, the miR-200 family, miR-148a, miR-193b, miR-126, and miR-205, have also been identified. miR-143 downregulates KRAS and ERK5 and represses the proliferation of metastatic prostate cancer PC-3M cells [114]. Some reports have indicated that immune cells release tumor-suppressive EVs. For example, NK cells release EVs containing perforin, CD56, and granzyme B to inhibit tumor growth [115]. However, in these EVs, only proteins, and not miRNAs, have been identified as effective components thus far. However, it is likely that immune cell-derived EVs also contain miRNAs, which contribute to suppress tumor progression.

## miRNAs in EVs as biomarkers for diagnostic, prognostic, and therapeutic targets

Extracellular miRNAs are anticipated to be useful as biomarkers because miRNAs in vesicles can be detected in body fluids (reviewed elsewhere, in [91, 116]), such as sera, urine, and saliva. In addition, EVs reflect different expression profiles in normal cells and cancerous cells in ovarian cancer [91], or cancer-derived exosomes selectively contain specific miRNAs in cells [73]. The profiling of miRNAs in EVs has become easier due to the development of microarray and next-generation deep-sequencing technologies [117].

### Diagnosis and prognosis

miRNAs found in EVs may be an indicator of diagnosis and prognosis. Many researchers have shown that the miRNA profiles in EVs are significantly different in patients with high-metastatic tumors. The miRNAs that may potentially be used as diagnostic and prognostic markers are summarized in [116]. Cancer-specific markers of EVs have been identified for application to cancer diagnostics at early stages. Microarray analyses revealed the accumulation of let-7a, miR-1229, miR-1246, miR-150, miR-21, miR-223, and miR-23a in the EV-concentrated fraction from patients with colorectal cancer [118]. In prostate cancer, miR-141 and miR-375 are promising markers of metastasis in the blood circulation. In particular, miR-141 levels were shown to be 46-fold higher in metastatic patients than healthy individuals in plasma [22, 119]. Both miRNAs were significantly increased in EVs in sera from men with metastatic disease [120] (reviewed in [121, 122]). EVs derived from patients with ovarian cancer were found to present a diagnostic profile of 8 miRNAs (miR-21, miR-141, miR-200a, miR-200c, miR-200b, miR-203, miR-205 and miR-214) [91]. In EVs from lung adenocarcinoma patients, the profiles of 12 miRNAs (miR-17-3p, miR-21, miR-106a, miR-146, miR-155, miR-191, miR-192, miR-203, miR-205, miR-210, miR-212, and miR-214), which are diagnostic in lung tumor biopsies, showed similar pattern [123]. The profiling of miRNAs in EVs is expected as a powerful strategy for cancer diagnosis and prognosis.

One bottleneck in the detection of miRNAs in EVs in the arena of diagnostics is the methods used for the collection of EVs. Collecting EVs using ultracentrifugation is a conventional way to collect EVs, but it is time consuming. Collecting EVs through filtration and affinity purification based on surface proteins are possible alternative strategies [124]. For expecting clinical use, EV collection using microfluidic immunoaffinity method that can directly bring into transcriptome analysis has been developed [125].

### Drug delivery systems

Extracellular vesicles are anticipated to be useful as a drug delivery system to deliver anti-tumor siRNAs, as nucleic acid medicines, specifically to cancer cells as a novel cancer therapy [126]. Although the native targeting system is not known, Ohno et al. engineered the surface of EVs to express the GE11 peptide or the EGF peptide, which is specifically recognized by EGFR. The engineered EVs were successfully delivered to EGFR-expressing breast cancer cells, and the encapsulated siRNAs repressed target gene expression [127]. Alvarez-Erviti et al. [128] also modified the surface of the exosome to deliver siRNA to brain in mice using self-derived exosomes from dendritic cells. For neuron-specific targeting, Lamp2b, and exosomal membrane protein, was fused to neuron-specific RVG peptide.

In addition, introducing exogenous siRNAs into exosome is another important aspect for drug delivery system. Wahlgren et al. [129] tested several methods to introduce double-stranded siRNA into plasma-derived exosome by electroporation and by chemical transfections using lipid micelles. They showed that lipid micelles themself transfer siRNA into cells and hard to distinguish from the delivery by exosome. Shtam et al. [130] also used both lipofectamine and electroporation to introduce siRNA into human exosomes. And they supported that electroporation is applicable for siRNA introduction into exosomes. Alvarez-Erviti et al. [128] used electroporation to introduce siRNA into exosomes. However, the efficiency of loading siRNAs by electroporation in exosome should be improved for drug delivery system, as the current efficiency is lower than 30 % [129].

Together with as-yet-undeveloped technologies for the targeted packaging of miRNAs, the targeting of EVs would be a useful technology in developing drug delivery systems for nucleic acid medicines.

### Therapeutics

As miRNAs transferred by cancer-derived EVs are known to promote cancer progression in many ways, the removal of cancer-secreted EVs is assumed to represent an effective means of repressing cancer proliferation and dissemination. Aethlon Medical Inc. has already developed a dialysis-like system that removes EVs in the circulating blood via affinity binding to immobilized EV-binding lectins and antibodies [131]. Although some concerns should be addressed before this system is used for treatment, such as how much impact the transient reduction of cancer-derived EVs has and whether the removal of EVs not only from cancer cells but also from non-cancerous cells in the body has a negative impact, this method is anticipated to serve as a novel cancer therapy.

## Conclusions and perspectives

MicroRNAs regulate gene expression levels post-transcriptionally. Not only do miRNAs function in the miRNA-coding cells, but they also appear to be functional in the recipient cells. miRNAs are secreted in a form protected by proteins or lipid bilayer vesicles, referred to as EVs. Increasing numbers of studies on EVs have revealed that EVs are an important cell–cell communication tool in biological activities and tumor progression.

EVs contain several molecules within one vesicle, carrying not only a single type of miRNA, but various types of miRNAs, mRNAs, proteins, and growth factors. The ability of EVs to transfer many types of molecules at the same time is a key feature of these vesicles compared with protein-bound miRNAs or other secreted biomolecules. In addition, cells are likely to take up multiple EVs simultaneously. Therefore, various types of molecules that they carry could have co-operative effects on the recipient cells. However, only a fraction of the miRNAs and molecules responsible for certain activities in the cells are known. To understand the true biological function of the roles of EVs, it will be necessary to evaluate the comprehensive effects of EVs on cells using different approaches.

miRNAs transferred by EVs function in tumor progression. Reducing cancer-derived EV uptake by inhibiting secretion and eliminating EVs would provide supportive therapeutic strategies to inhibit cancer progression and metastasis. In addition, enhancing or mimicking cancer-suppressive EVs could also be employed as a novel therapy.
